# Sequentially Estimating the Approximate Conditional Mean Using Extreme Learning Machines

**DOI:** 10.3390/e22111294

**Published:** 2020-11-13

**Authors:** Lijuan Huo, Jin Seo Cho

**Affiliations:** 1 School of Humanities and Social Sciences, Beijing Institute of Technology, Haidian, Beijing 100081, China; ljhuo@bit.edu.cn; 2 School of Economics, Yonsei University, Seodaemun, Seoul 03722, Korea

**Keywords:** conditional mean specification testing, omnibus test, gaussian process, extreme learning machine, wald test statistic, functional regression, sequential testing procedure, consistent correct model estimation

## Abstract

This study examined the extreme learning machine (ELM) applied to the Wald test statistic for the model specification of the conditional mean, which we call the WELM testing procedure. The omnibus test statistics available in the literature weakly converge to a Gaussian stochastic process under the null that the model is correct, and this makes their application inconvenient. By contrast, the WELM testing procedure is straightforwardly applicable when detecting model misspecification. We applied the WELM testing procedure to the sequential testing procedure formed by a set of polynomial models and estimate an approximate conditional expectation. We then conducted extensive Monte Carlo experiments to evaluate the performance of the sequential WELM testing procedure and verify that it consistently estimates the most parsimonious conditional mean when the set of polynomial models contains a correctly specified model. Otherwise, it consistently rejects all the models in the set.

## 1. Introduction

Conducting data inference using correctly specified models is desirable for predicting future observations. If models are misspecified, however, proper data inference cannot be conducted, and predicting future observations may then involve an undesired bias. Because of this, previous studies have developed methodologies to test the correct model assumptions. For example, in a classical study, Ramsey [1] provides a test statistic for non-linearity. In another classical study, Bierens [2] provides an omnibus model specification test statistic that detects arbitrary model misspecification consistently. In addition to these works, a number of studies provide correct model specification testing methodologies [3,4,5,6,7].

Despite the rapid development of correct model specification testing, researchers may still be unable to obtain a correctly specified model and may have to predict future observations using misspecified models. If all candidate models are misspecified by model specification tests, the model with the lowest mean square error is typically chosen to forecast future observations, even if it is known to be misspecified.

To address this concern, the present study provides a robust methodology to search for a correct model in a systematic way. To do so, we developed a sequential testing procedure that combines the model specification test statistic available in the previous literature with high-degree polynomial models, so that a close approximation of the conditional mean equation can be consistently estimated.

In previous studies, model specification testing using artificial neural networks (ANNs) are widely applied because of their universal approximation property [8,9,10]. Cho, Ishida, and White [7] propose an ANN-based quasi-likelihood ratio (QLR) statistic for testing neglected non-linearity that exploits the generically comprehensively revealing (GCR) feature of the ANN-based test statistic and overcomes the so-called twofold Davies’ [11,12] identification problem to obtain its null limit distribution as a functional of a Gaussian stochastic process.

However, despite the theoretical efficacy of the QLR test statistic, it may not be convenient for empirical applications. Its null limit distribution is dependent on the model scopes, so that the asymptotic critical values are different from model to model. Cho, Phillips, and Seo [13] and Cho, Huang, and White [14] note this inconvenience and define a Wald test statistic using a functional regression, so that it follows a chi-squared distribution under the null hypothesis that the model is correctly specified. Cho and White [15] further demonstrate that, if the extreme learning machine (ELM) proposed by Huang, Zhu, and Siew [16] is combined with the Wald test statistic, its computation can be efficiently performed in addition to being GCR. They refer to this as the Wald-ELM (WELM) testing procedure.

The polynomial model is also widely applied for empirical applications. Its popularity lies in the fact that it has a recursive structure and can uniformly approximate any continuous function. This aspect makes it convenient to apply it to a sequential testing procedure. If a lower-degree polynomial model is rejected by a proper testing procedure, we can consider its next higher-degree polynomial model as another approximation and test model adequacy.

Previous studies also apply sequential testing procedures to polynomial models. Cho and Phillips [17] develop a sequential testing methodology to test the null of a polynomial function to identify the polynomial degree by extending the testing methodology of Baek, Cho, and Phillips [18]. Specifically, Baek, Cho, and Phillips [18] note that, if the QLR test statistic in Cho, Ishida, and White [7] is applied to a linear model augmented by a power transformation, the twofold identification problem is transformed into a trifold Davies’ [11,12] identification problem. They overcome this and derive the null limit distribution of the QLR test statistic. Hence, they recommend using Hansen’s [19] weighted bootstrap for empirical applications because the null limit distribution is associated with a Gaussian stochastic process as in the twofold identification problem. Specifically, the null limit distribution is represented by the maximum of the squared Gaussian process, so that the asymptotic critical values are different from model to model, making its application inconvenient for obtaining asymptotic critical values. Cho and Phillips [17] extend the QLR test statistic to test the null of the polynomial function hypothesis and obtain its null limit distribution by overcoming the multifold identification problem, which is further developed from the trifold identification problem in Baek, Cho, and Phillips [18]. In addition to this derivation, they apply the null limit distribution to the sequential testing procedure to search for a close approximation of the conditional mean function. For practical applications of the sequential testing procedure, they also recommend applying the weighted bootstrap as in Baek, Cho, and Phillips [18].

In this study, we applied the WELM test statistic to the sequential testing procedure. This statistic is convenient for applications, as well as possesses the GCR feature, so that it can be employed in this study. In addition, the WELM test statistic has features not shared by the test statistics used in the literature. First, the null limit distribution is obtained as a chi-squared distribution, so that traditional theory on the sequential testing procedure can be applied [20]. Hence, we do not need to apply approximation theory on the probability of the maximum of a squared Gaussian process as for the QLR test statistic. Furthermore, as we discuss below, the sequential testing procedure is conducted by reducing the level of significance in response to a rise in the sample size, so that the degree estimation error reduces to zero asymptotically. As the null limit distribution is chi-squared, we can easily choose the plans for the level of significance without satisfying the additional condition for the application of the QLR test statistic that the level of significance slowly converges to zero. This condition does not have to be imposed in our sequential testing procedure. Second, the conditioning variable does not have to be positively valued as required by Cho and Phillips [15]. Even when the conditioning variable is negatively valued, the sequential testing procedure using the WELM test statistic is directly applicable.

The rest of this paper is organized as follows. Section 2 focuses on the polynomial model and provides the null limit distribution of the WELM test statistic, along with a literature review. Section 3 applies the WELM test statistic to the sequential testing procedure and provides the theoretical results. Section 4 discusses the extensive simulations conducted using the WELM test statistic and sequential testing procedure. We consider three data-generating processes (DGPs) and examine how the sequential testing procedure responds to various plans for the level of significance. Section 5 provides concluding remarks and summarizes the main findings. All the mathematical proofs are presented in the Appendix A.

## 2. Method 1: Application of the WELM Test to the Polynomial Model

In this section, we first describe the main motivation of this study in relation to the development of the literature in terms of model specification testing. To fix our idea, we focus on the WELM test statistic applied to the polynomial model. Our primary interest is in developing a statistical methodology to estimate the conditional mean equation of time-series observations. We therefore suppose that data are weakly dependent observations as follows:

**Assumption** **1 (DGP).** *Let (Ω,F,P) be a complete probability space and let k∈N. Let $\left\{{\left(y_{t},x_{t},d_{t}^{\prime }\right)}^{\prime }:\Omega \mapsto R^{2+k}:t=1,2,\ldots \right\}$ be a strictly stationary and absolutely regular process with mixing coefficients $\beta_{\tau }$ such that for some ρ>1, $\sum_{t=1}^{\infty }\tau^{2\rho /(\rho -1)}\beta_{\tau }<\infty$ and $x_{t}$ is strictly non-negative with probability 1.*

Here, $y_{t}$ and $z_{t}:={\left(x_{t},d_{t}^{\prime }\right)}^{\prime }$ are serially dependent target and explanatory variables, respectively, and $z_{t}$ can contain the lagged target variables, so that dynamic misspecification can be removed from our consideration. Specifically, researchers are concerned about possible non-linearity with respect to $x_{t}$ when they attempt to approximate the conditional mean equation using the *p*-th-degree polynomial function:

$$E\left[y_{t}|F_{t}\right]\approx x_{t}{\left(p\right)}^{\prime }\alpha_{*}\left(p\right)+d_{t}^{\prime }\eta_{*},$$

where $x_{t}\left(p\right):={\left(1,x_{t},\ldots ,x_{t}^{p}\right)}^{\prime }$, $\theta_{*}\left(p\right):={\left(\alpha_{*}{\left(p\right)}^{\prime },\eta_{*}\right)}^{\prime }$ is the linear coefficient of ${\left(x{\left(p\right)}^{\prime },d_{t}^{\prime }\right)}^{\prime }$, and $F_{t}$ is the smallest σ-field generated by $\left(z_{t},y_{t-1},z_{t-1},y_{t-2},\ldots \right)$.

The polynomial functions are uniformly dense and this motivates us to estimate the conditional mean using the above specification. The Stone-Weierstrass theorem implies that continuous functions are uniformly approximated by polynomial functions with high levels of degrees, so that the above polynomial function becomes a successful approximation of the conditional mean if the degree *p* is sufficiently large.

The current study seeks to provide a statistical method to estimate the degree of the polynomial function in the most parsimonious manner. The non-local behavior of a high-degree polynomial model is understood as one of the drawbacks of estimating the high-degree polynomial model using regression. That is, the outlier of $x_{t}$ can substantially affect the estimated forecast, and this can reduce the utility of the polynomial model estimation [21].

We accommodate this aspect by estimating the polynomial using the most parsimonious model. Specifically, we estimate the polynomial degree *p* as small as possible, and for this purpose, we provide a sequential testing methodology described in the next section. In particular, our testing approach is based upon the GCR property of an ANN model and ELMs.

To describe our testing procedure using the ELM applied to the GCR property, we note that Stinchcombe and White [10] show that when the regression model is estimated by attaching an analytic function to a linear model, the linear coefficient consistently estimates a non-zero coefficient if and only if the regression model is misspecified for the conditional mean equation; the authors refer to this as the GCR property. Specifically, the following assumption gives the model advocated by Stinchcombe and White [10]:

**Assumption** **2 (Model).** *Let $M_{p}:=\left\{f\left(\cdot ;\theta \left(p\right),\lambda ,\delta \right):\left(\theta \left(p\right),\lambda ,\delta \right)\in \Theta \left(p\right)\times \Lambda \times \Delta \right\}$ be specified as the alternative model, where*

$$f\left(z_{t};\theta \left(p\right),\lambda ,\delta \right):=x_{t}{\left(p\right)}^{\prime }\alpha \left(p\right)+d_{t}^{\prime }\eta +\lambda \Psi \left(\delta x_{t}\right)$$

*and Ψ(·), δ, and λ, are the additional hidden unit constructed by an analytic function, input-to-hidden weight, and hidden-to-output weight, respectively.*

Here, if we let $\lambda_{*}$ be the probability limit of the parameter estimated by regression, the GCR property implies that the estimated coefficient for $\lambda_{*}$ is consistently different from zero if the *p*-th-degree polynomial model is misspecified for the conditional mean. Therefore, we can detect whether the *p*-th-degree polynomial model is correct by testing whether the coefficient of the hidden unit is zero. That is, if the estimated hidden-to-output weight is statistically different from zero, it means that $M_{p}$ does not approximate the model sufficiently well. Otherwise, $M_{p}$ becomes a successful approximation of the conditional mean, motivating us to rephrase the following hypotheses:

$$H_{0}:\mathrm{For}\;\mathrm{some}\;\theta_{*}\left(p\right)\in \Theta \left(p\right),\;P\left[E\left(y_{t}|F_{t}\right)=x_{t}{\left(p\right)}^{\prime }\alpha_{*}\left(p\right)+d_{t}^{\prime }\eta_{*}\right]=1\;\;\;\;\mathrm{versus}$$

$$H_{1}:\mathrm{For}\;\mathrm{every}\;\theta \left(p\right)\in \Theta \left(p\right),\;P\left[E\left(y_{t}|F_{t}\right)=x_{t}{\left(p\right)}^{\prime }\alpha \left(p\right)+d_{t}^{\prime }\eta \right]<1$$

into the following equivalent hypotheses:

$$H_{0}^{\prime }:\lambda_{*}=0\;\;\;\mathrm{versus}\;\;\;\;H_{1}^{\prime }:\lambda_{*}\neq 0$$

in their framework. This implies that we can let our null model be

$$M_{p}^{0}:=\left\{f^{0}\left(\cdot ;\theta \left(p\right)\right):\theta \left(p\right)\in \Theta \left(p\right)\right\}$$

and $f^{0}\left(z_{t};\theta \left(p\right)\right):=x_{t}{\left(p\right)}^{\prime }\alpha \left(p\right)+d_{t}^{\prime }\eta$ for p=1,2,…. In what follows, we let $\Psi_{t}\left(\delta \right)$ denote $\Psi (\delta x_{t})$ for notational simplicity.

This aspect now implies that the GCR property can be exploited by testing $H_{0}^{\prime }$ against $H_{1}^{\prime }$, and we need to test whether the input-to-output weight is zero.

We now provide the regularity conditions for the regular behavior of the test statistics provided below:

**Assumption** **3 (Regularity).** *(i) (Δ,D,Q) and (Ω×Δ,F×D,P·Q) are complete probability spaces. (ii) For p∈N, Θ(p) is a non-empty compact and convex set, and *Λ* and *Δ* are non-empty compact and convex subsets such that 0 is an interior element of *Λ*. (iii) For p∈N, $\sum_{t=1}^{n}w_{t}\left(p\right)w_{t}{\left(p\right)}^{\prime }$ is positive definite with probability 1 and $E[w_{t}\left(p\right)w_{t}{\left(p\right)}^{\prime }]$ is positive definite, where $w_{t}\left(p\right):={\left(x_{t}{\left(p\right)}^{\prime },d_{t}^{\prime }\right)}^{\prime }$. (iv) Ψ:R↦R is a non-polynomial analytic function. (v) $E[y_{t}^{2}]<\infty$, $E[x_{t}^{2p}]<\infty$, and there is a sequence of stationary and ergodic random variables $\left\{s_{t}\right\}$ such that (v.a) $|u_{t}|\leq s_{t}$, (v.b) $\sup_{\delta \in \Delta }\left|\Psi \left(\delta x_{t}\right)\right|\leq s_{t}$, (v.c) ${\left(\int_{\Delta }\Psi_{t}\left(\delta \right)dQ\left(\delta \right)\right)}^{2}\leq s_{t}$, (v.d) $\sup_{\delta \in \Delta }\left|\left(\partial \Psi \left(\delta x_{t}\right)\right)/\left(\partial \delta \right)\right|\leq s_{t}$, and (v.e) for some κ≥4ρ, $E[|s_{t}{|}^{\kappa }]<\infty$.*

Assumptions 1–3 are obtained by adapting the regularity conditions in Cho and White [15] to the current polynomial model structure. Their model assumes non-linearity with respect to the parameters, and we further simplify their assumptions by imposing the polynomial model structure used herein, so that the limit results provided below can be obtained as corollaries of their theorems.

Indeed, testing $H_{0}^{\prime }:\lambda_{*}=0$ is irregular because it involves Davies’ [11,12] identification problem. That is, if $\lambda_{*}=0$, $\delta_{*}$ is not identified, $\delta_{*}$ is identified only when $\lambda_{*}\neq 0$, so that the null limit distribution of the *t*-test statistic testing $H_{0}^{\prime }$ becomes different from the standard normal distribution. The null limit distribution is found to be characterized by a Gaussian stochastic process indexed by the unidentified parameter δ. That is, if we let $t_{n}$ be the standard *t*-test statistic testing $H_{0}^{\prime }$, it follows that

$$t_{n}\Rightarrow \underset{\delta \in \Delta }{\sup}G\left(\delta \right)$$

under $H_{0}^{\prime }$ and Assumptions 1–3, where G(·) is a Gaussian stochastic process such that for every δ∈Δ, E[G(δ)]=0, and for each $\left(\delta ,\delta^{\prime }\right)$,

$$E\left[G\left(\delta \right)G\left(\delta^{\prime }\right)\right]=\frac{\rho (\delta ,\delta^{\prime })}{{\left\{r\left(\delta ,\delta \right)\right\}}^{1/2}{\left\{r\left(\delta^{\prime },\delta^{\prime }\right)\right\}}^{1/2}}$$

with

$$\rho \left(\delta ,\delta^{\prime }\right)=E\left[u_{t}^{2}\Psi_{t}^{*}\left(\delta \right)\Psi_{t}^{*}\left(\delta^{\prime }\right)\right]\;\;\;\;\mathrm{and}\;\;\;\;r\left(\delta ,\delta^{\prime }\right)=E\left[u_{t}^{2}\right]E\left[\Psi_{t}^{*}\left(\delta \right)\Psi_{t}^{*}\left(\delta^{\prime }\right)\right].$$

Here, we let $\Psi_{t}^{*}\left(\delta \right):=\Psi_{t}\left(\delta \right)-E\left[\Psi_{t}\left(\delta \right)w_{t}{\left(p\right)}^{\prime }\right]E{\left[w_{t}\left(p\right)w_{t}{\left(p\right)}^{\prime }\right]}^{-1}w_{t}\left(p\right)$, and $u_{t}:=y_{t}-E\left[y_{t}|F_{t}\right]$.

This limit distribution makes it inconvenient to apply the standard *t*-test statistic when testing $H_{0}^{\prime }$ against $H_{1}^{\prime }$. The limit distribution is affected by too many factors in terms of the data and model. If the error $u_{t}$ is conditionally homoscedastic, the associated Gaussian process is a standard Gaussian process in the sense that for every δ∈Δ, G(δ)∼N(0,1). However, this does not hold if $u_{t}$ is conditionally heteroscedastic. Furthermore, there are many candidate analytic functions for Ψ(·). As Cho and White [7] highlight, the previous literature chooses different functions for Ψ(·), namely the logistic cumulative distribution function in White [22], exponential function in Bierens [2] and ridgelet function in Candés [23], among others. Different covariance kernel structures are obtained for the different analytic functions selected for Ψ(·), and this leads to different null limit distributions for the *t*-test statistic. Empirical researchers applying the standard *t*-test statistic have to apply different critical values to different models and data, making it more difficult to obtain asymptotic critical values than the test statistic value itself. This aspect also analogously applies to other standard test statistics, such as Wald, Lagrange multiplier, and QLR.

To overcome this, we use another testing method that applies the ELM proposed by Huang, Zhu, and Siew [16]. Cho and White [15] note that the functional ordinary least squares (FOLS) estimator suggested by Cho, Huang, and White [14] and Cho, Phillips, and Seo [13] can be exploited to yield a straightforward statistic to test $H_{0}^{\prime }$ against $H_{1}^{\prime }$ by applying the ELM. As we detail below, the FOLS estimator has a limit distribution involved with integration, which lets the estimator follow a normal distribution asymptotically instead of being characterized by the Gaussian process. Using this property, we can convert the FOLS estimator into a Wald test statistic to follow a chi-squared distribution asymptotically under the null hypothesis. Here, the ELMs are exploited to compute the involved integrations.

Specifically, first, for each δ, $E[u_{t}\Psi_{t}\left(\delta \right)]=0$ under $H_{0}$ because $\Psi_{t}\left(\delta \right):=\Psi \left(\delta x_{t}\right)$ is measurable with respect to $F_{t}$ and $u_{t}$ is a martingale difference sequence from the fact that $u_{t}:=y_{t}-E\left[y_{t}|F_{t}\right]$, so that $E[u_{t}|F_{t}]=0$. This implies that, if $\Psi_{t}\left(\delta \right)$ is regressed against $\left(1,u_{t}\right)$, the estimated coefficient of $u_{t}$ has to be zero irrespective of δ. Therefore, instead of testing $H_{0}^{\prime }$ against $H_{1}^{\prime }$, we opt to test

$$H_{0}^{\prime \prime }:\beta_{*}\left(\cdot \right)\equiv 0\;\;\;\;\;\mathrm{vs}\;\;\;\;\;H_{1}^{\prime \prime }:\beta_{*}\left(\cdot \right)\neq 0,$$

where for each δ∈Δ,

$$\left[\begin{array}{c} \alpha_{*}\left(\delta \right)\\ \beta_{*}\left(\delta \right) \end{array}\right]:={\left[\begin{array}{cc} 1 & E[u_{t}]\\ E[u_{t}] & E[u_{t}^{2}] \end{array}\right]}^{-1}\left[\begin{array}{c} E[\Psi_{t}\left(\delta \right)]\\ E[u_{t}\Psi_{t}\left(\delta \right)] \end{array}\right].$$

Here, $E[u_{t}]=0$ and thus $u_{t}$ is a martingale difference sequence. Nevertheless, many of the entities on the right side are unknown to the researcher, necessitating the estimation of each expectation by its sample analog: for each δ∈Δ,

$$\left[\begin{array}{c} {\hat{\alpha }}_{n}\left(\delta \right)\\ {\hat{\beta }}_{n}\left(\delta \right) \end{array}\right]:={\left[\begin{array}{cc} 1 & \sum_{t=1}^{n}{\hat{u}}_{t}\\ \sum_{t=1}^{n}{\hat{u}}_{t} & \sum_{t=1}^{n}{\hat{u}}_{t}^{2} \end{array}\right]}^{-1}\left[\begin{array}{c} \sum_{t=1}^{n}\Psi_{t}\left(\delta \right)\\ \sum_{t=1}^{n}{\hat{u}}_{t}\Psi_{t}\left(\delta \right) \end{array}\right],$$

where ${\hat{u}}_{t}$ is the regression residual obtained from $M_{p}^{0}$, namely

$${\hat{u}}_{t}:=y_{t}-w_{t}{\left(p\right)}^{\prime }{\left(\sum_{t=1}^{n}w_{t}\left(p\right)w_{t}{\left(p\right)}^{\prime }\right)}^{-1}\sum_{t=1}^{n}w_{t}\left(p\right)y_{t},$$

so that it also follows that $\sum_{t=1}^{n}{\hat{u}}_{t}\equiv 0$ and consistently estimates $u_{t}$ under $H_{0}$. As there are a continuum of δs in Δ, Cho and White [15] integrate the above estimators using an adjunct probability measure Q(·) and obtain the following limit distribution:

(1) $$\left[\begin{array}{c} \sqrt{n}\int_{\Delta }\left({\hat{\alpha }}_{n}\left(\delta \right)-\alpha_{*}\left(\delta \right)\right)dQ\left(\delta \right)\\ \sqrt{n}\int_{\Delta }{\hat{\beta }}_{n}\left(\delta \right)dQ\left(\delta \right) \end{array}\right]\Rightarrow {\left[\begin{array}{cc} 1 & 0\\ 0 & E[u_{t}^{2}] \end{array}\right]}^{-1}\left[\begin{array}{c} \int_{\Delta }G_{1}\left(\delta \right)dQ\left(\delta \right)\\ \int_{\Delta }G_{2}\left(\delta \right)dQ\left(\delta \right) \end{array}\right]$$

under $H_{0}$ and Assumptions 1–3, where Q(·) is an adjunct probability measure defined on Δ (which is selected by the researcher), and $G_{1}\left(\cdot \right)$ and $G_{2}\left(\cdot \right)$ are two independent Gaussian processes such that for each δ∈Δ, $E[G_{1}\left(\delta \right)]=0$ and $E[G_{2}\left(\delta \right)]=0$, and for each δ and $\delta^{\prime }\in \Delta$,

$$E\left[G_{1}\left(\delta \right)G_{1}\left(\delta^{\prime }\right)\right]=\tau \left(\delta ,\delta^{\prime }\right):=E\left[\Psi_{t}\left(\delta \right)\Psi_{t}\left(\delta^{\prime }\right)\right]-E\left[\Psi_{t}\left(\delta \right)\right]E\left[\Psi_{t}\left(\delta^{\prime }\right)\right],\;\;\;\mathrm{and}\;\;\;E\left[G_{2}\left(\delta \right)G_{2}\left(\delta^{\prime }\right)\right]=\rho \left(\delta ,\delta^{\prime }\right).$$

This null limit distribution is indeed obtained by following the limit distribution theory of the FOLS estimator in Cho, Huang, and White [14] and Cho, Phillips, and Seo [13], in which they test the population mean function of functional data by estimating a parametric model using the FOLS estimator. More precisely, the FOLS estimator is obtained by minimizing the following functional mean squared errors:

$$Q_{n}\left(\gamma ,\xi \right):=\frac{1}{2n}\sum_{t=1}^{n}\int_{\Delta }{\left(\Psi_{t}\left(\delta \right)-\gamma -\xi {\hat{u}}_{t}\right)}^{2}dQ\left(\delta \right)$$

with respect to γ and ξ. If we let $\left({\hat{\gamma }}_{n},{\hat{\xi }}_{n}\right)$ denote the FOLS estimator minimizing $Q_{n}\left(\cdot ,\cdot \right)$, it now follows that

$$\begin{array}{cc} \left[\begin{array}{c} {\hat{\gamma }}_{n}\\ {\hat{\xi }}_{n} \end{array}\right]={\left[\begin{array}{cc} 1 & \sum_{t=1}^{n}{\hat{u}}_{t}\\ \sum_{t=1}^{n}{\hat{u}}_{t} & \sum_{t=1}^{n}{\hat{u}}_{t}^{2} \end{array}\right]}^{-1} & \left[\begin{array}{c} \sum_{t=1}^{n}\int_{\Delta }\Psi_{t}\left(\delta \right)dQ\left(\delta \right)\\ \sum_{t=1}^{n}\int_{\Delta }{\hat{u}}_{t}\Psi_{t}\left(\delta \right)dQ\left(\delta \right) \end{array}\right]\\ \; & \overset{a.s.}{\to }\left[\begin{array}{c} \gamma_{*}\\ \xi_{*} \end{array}\right]:={\left[\begin{array}{cc} 1 & 0\\ 0 & E[u_{t}^{2}] \end{array}\right]}^{-1}\left[\begin{array}{c} \int_{\Delta }E\left[\Psi_{t}\left(\delta \right)\right]dQ\left(\delta \right)\\ \int_{\Delta }E\left[u_{t}\Psi_{t}\left(\delta \right)\right]dQ\left(\delta \right) \end{array}\right] \end{array}$$

under Assumptions 1–3, leading to that

$$\left[\begin{array}{c} \sqrt{n}\left({\hat{\gamma }}_{n}-\gamma_{*}\right)\\ \sqrt{n}\left({\hat{\xi }}_{n}-\xi_{*}\right) \end{array}\right]\Rightarrow {\left[\begin{array}{cc} 1 & 0\\ 0 & E[u_{t}^{2}] \end{array}\right]}^{-1}\left[\begin{array}{c} \int_{\Delta }G_{1}\left(\delta \right)dQ\left(\delta \right)\\ \int_{\Delta }G_{2}\left(\delta \right)dQ\left(\delta \right) \end{array}\right].$$

This limit distribution is now identical to that in (1), and $\xi_{*}=0$ under $H_{0}$.

Based on the FOLS estimator, Cho and White [15] test the null hypothesis using the Wald test statistic. Integrating Gaussian processes produces a normally distributed random variable, implying that

$$\left[\begin{array}{c} \int_{\Delta }G_{1}\left(\delta \right)dQ\left(\delta \right)\\ \int_{\Delta }G_{2}\left(\delta \right)dQ\left(\delta \right) \end{array}\right]∼N\left[\left(\begin{array}{c} 0\\ 0 \end{array}\right),\left(\begin{array}{cc} \sigma_{\gamma }^{2} & 0\\ 0 & \sigma_{\xi }^{2} \end{array}\right)\right],$$

where

$$\sigma_{\gamma }^{2}:=\int_{\Delta }\int_{\Delta }\tau \left(\delta ,\delta^{\prime }\right)dQ\left(\delta \right)dQ\left(\delta^{\prime }\right)\;\;\;\;\;\mathrm{and}\;\;\;\;\sigma_{\xi }^{2}:=\int_{\Delta }\int_{\Delta }\rho \left(\delta ,\delta^{\prime }\right)dQ\left(\delta \right)dQ\left(\delta^{\prime }\right).$$

This further implies that

(2) $$\left[\begin{array}{c} \sqrt{n}\int_{\Delta }\left({\hat{\alpha }}_{n}\left(\delta \right)-\alpha_{*}\left(\delta \right)\right)dQ\left(\delta \right)\\ \sqrt{n}\int_{\Delta }{\hat{\beta }}_{n}\left(\delta \right)dQ\left(\delta \right) \end{array}\right]\overset{A}{∼}N\left[\left(\begin{array}{c} 0\\ 0 \end{array}\right),\left(\begin{array}{cc} \sigma_{\gamma }^{2} & 0\\ 0 & \sigma_{\xi }^{2}/\sigma_{u}^{2} \end{array}\right)\right]$$

under $H_{0}$, where $\sigma_{u}^{2}:=E\left[u_{t}^{2}\right]$, and the null limit distribution of the FOLS estimator now motivates us to construct the following Wald test statistic:

$$W_{n}:=n\left(\frac{{\hat{\sigma }}_{u,n}^{2}}{{\hat{\sigma }}_{\xi ,n}^{2}}\right){\left(\int_{\Delta }{\hat{\beta }}_{n}\left(\delta \right)dQ\left(\delta \right)\right)}^{2},$$

which follows a chi-squared distribution with one degree of freedom under $H_{0}^{\prime \prime }$ and Assumptions 1–3, where ${\hat{\sigma }}_{\xi ,n}^{2}$ and ${\hat{\sigma }}_{u,n}^{2}$ are consistent estimators of $\sigma_{\xi }^{2}$ and $\sigma_{u}^{2}$, respectively. Under $H_{1}^{\prime \prime }$, $\int_{\Delta }\beta_{*}\left(\delta \right)dQ\left(\delta \right)$ is not necessarily equal to zero, and we can expect power for this test statistic from this aspect. The test statistic is defined following Wald’s [24] test principle. Owing to its trivial null limit behavior, its empirical applicability is more straightforward than other test statistics requiring extra efforts by the researcher to obtain the asymptotic critical values, namely the QLR test statistic in Baek, Cho, and Phillips [18] and Cho and Phillips [17].

Nevertheless, the burden of computing the Wald test statistic can be immense because of the involved integrations. To compute the statistic, it is thus necessary to calculate the integration of $\Psi_{t}\left(\cdot \right)$ for each *t*, and if *n* is large, the involved computational burden can be huge.

Cho and White [15] recommend resolving this issue by applying the ELM proposed by Huang, Zhu, and Siew [16]. That is, if we let $\left\{\delta_{i}:i=1,2,\ldots ,m\right\}$ be a set of identically and independently distributed (IID) random variables following the Q distribution, it follows that

$${\bar{\Psi }}_{t,m}:=\frac{1}{m}\sum_{i=1}^{m}\Psi_{t}\left(\delta_{i}\right)\overset{a.s.}{\to }\int_{\Delta }\Psi_{t}\left(\delta \right)dQ\left(\delta \right)$$

by the law of large numbers, so that the FOLS estimator can be well approximated by

$$\left[\begin{array}{c} {\hat{\gamma }}_{m,n}\\ {\hat{\xi }}_{m,n} \end{array}\right]:={\left[\begin{array}{cc} 1 & \sum_{t=1}^{n}{\hat{u}}_{t}\\ \sum_{t=1}^{n}{\hat{u}}_{t} & \sum_{t=1}^{n}{\hat{u}}_{t}^{2} \end{array}\right]}^{-1}\left[\begin{array}{c} \sum_{t=1}^{n}{\bar{\Psi }}_{t,m}\\ \sum_{t=1}^{n}{\hat{u}}_{t}{\bar{\Psi }}_{t,m} \end{array}\right]$$

if *m* is sufficiently large. To implement this plan, we formally assume the following condition.

**Assumption** **4 (ELM).** *$\left\{\delta_{j}\right\}$ is a sequence of IID random variables defined on (Δ,D,Q).*

Then, we can expect that

$$W_{m,n}:=n{\hat{\sigma }}_{n}^{2}\left(\frac{{\hat{\xi }}_{m,n}^{2}}{{\hat{\sigma }}_{\xi ,m,n}^{2}}\right)\overset{A}{∼}X_{1}^{2}$$

under $H_{0}^{\prime \prime \prime }$ and Assumptions 1–4, where

$${\hat{\sigma }}_{\xi ,m,n}^{2}:=\frac{1}{n}\sum_{t=1}^{n}{\hat{u}}_{t}^{2}{\bar{\Psi }}_{t,m,n}^{*2}\;\;\;\mathrm{and}$$

$${\bar{\Psi }}_{t,m,n}^{*}:={\bar{\Psi }}_{t,m}-\left(\frac{1}{n}\sum_{t=1}^{n}{\bar{\Psi }}_{t,m}w_{t}\left(p\right)\right){\left(\frac{1}{n}\sum_{t=1}^{n}w_{t}\left(p\right)w_{t}{\left(p\right)}^{\prime }\right)}^{-1}w_{t}\left(p\right).$$

The only difference between $W_{n}$ and $W_{m,n}$ is in the fact that ${\hat{\xi }}_{m,n}$ is used to estimate $\int_{\Delta }{\hat{\beta }}_{n}\left(\delta \right)dQ\left(\delta \right)$. Following Cho and White [15], we also refer to this as the WELM test statistic.

Cho and White [15] show by simulation that the null distribution of the WELM test statistic is well approximated by the chi-squared distribution by letting *n* and *m* be sufficiently large when their null model is the first-order autoregressive model. In addition, they verify that the WELM test statistic displays respectful power.

Before moving onto the next section, we collect the main claims in this section into the following lemma.

**Lemma** **1.** Given Assumptions 1–4, (i) $W_{n}\overset{A}{∼}X_{1}^{2}$ under $H_{0}^{\prime \prime }$; and for any positive sequence $\left\{c_{n}\right\}$ such that $c_{n}=o\left(n\right)$, if $\int_{\Delta }\beta_{*}\left(\delta \right)dQ\left(\delta \right)\neq 0$, $P(W_{n}>c_{n})\to 1$ under $H_{1}^{\prime \prime }$; and (ii) $W_{m,n}\overset{A}{∼}X_{1}^{2}$ under $H_{0}^{\prime \prime }$ as m and n→∞; and for any positive sequence $\left\{c_{n}\right\}$ such that $c_{n}=o\left(n\right)$, $P(W_{m,n}>c_{n})\to 1$ under $H_{1}^{\prime \prime }$ as m and n→∞.

Lemma 1(*i* and *ii*) are provided by Cho, Huang, and White [14] and Cho and White [15], respectively, in a general context, but we provide their proofs in the Appendix A to fit the current context.

## 3. Method 2: Sequential WELM Testing Procedure

In this section, we examine the sequential testing procedure combined with the WELM test statistic. The WELM test statistic developed by Cho and White [15] focuses on specification testing. We develop a testing methodology to estimate the most parsimonious polynomial model by combining the WELM test statistic with a sequential testing procedure.

To fix our idea on the sequential testing procedure, we first provide our model. The model in Assumption 2 assumes a *p*-th-degree polynomial model, and we now suppose that there are $\bar{p}$ polynomial models altogether:

$$M\left(\bar{p}\right):=\left\{M_{p}:p=1,2,\ldots ,\bar{p}\right\}\;\;\;\;\mathrm{and}\;\;\;\;M^{0}\left(\bar{p}\right):=\left\{M_{p}^{0}:p=1,2,\ldots ,\bar{p}\right\},$$

so that $M(\bar{p})$ and $M^{0}\left(\bar{p}\right)$ are the sets of the alternative and null models, respectively. These model sets encompass the models in Assumption 2 as special cases. That is, $M_{p}$ and $M_{p}^{0}$ in Assumption 2 are elements of $M(\bar{p})$ and $M^{0}\left(\bar{p}\right)$, respectively.

The most parsimonious model, which we seek to estimate using a sequential testing procedure, is obtained by testing smaller models against larger models sequentially. Specifically, the following procedure is proposed as our sequential testing procedure:

**Step** **1:** We test $M_{1}^{0}$ against $M_{1}$ using the WELM test statistic. If $M_{1}^{0}$ cannot be rejected at the level of significance α, we stop the sequential testing procedure and conclude that the conditional mean is linear with respect to $x_{t}$. Otherwise, we move onto the next step. The regression residual is computed by regressing $y_{t}$ on $\left(1,x_{t}\right)$ when computing the WELM test statistic.
**Step** **2:** We test $M_{2}^{0}$ against $M_{2}$ using the WELM test statistic. If $M_{2}^{0}$ cannot be rejected at the level of significance α, we stop the sequential testing procedure; otherwise, we move onto the next step. In this way, we continue our testing procedure until we reach $p=\bar{p}$. As in the first step, the regression residual is computed by regressing $y_{t}$ on $\left(1,x_{t},\ldots ,x_{t}^{p}\right)$ to compute the WELM statistic, which tests $M_{p}^{0}$ against $M_{p}$ for $p=2,3,\ldots ,\bar{p}-1$.
**Step** **3:** We test $M_{\bar{p}}^{0}$ against $M_{\bar{p}}$ using the WELM test statistic. If $M_{\bar{p}}^{0}$ cannot be rejected, we stop the sequential testing procedure to conclude that $E[y_{t}|F_{t}]$ is sufficiently well approximated by $M_{\bar{p}}^{0}$; otherwise, we conclude that $M^{0}\left(\bar{p}\right)$ is entirely misspecified for $E[y_{t}|F_{t}]$.

Using this procedure, the most parsimonious and correct model is consistently detected. For a specific discussion, for some $\alpha_{*}\left(p\right)$ and $\eta_{*}$, let $p_{*}$ be defined as

$$p_{*}:=\min\left\{p\in N:E\left[y_{t}|F_{t}\right]=x_{t}{\left(p\right)}^{\prime }\alpha_{*}\left(p\right)+d_{t}^{\prime }\eta_{*}\right\}.$$

Note that $p_{*}$ is the smallest polynomial degree such that the conditional mean is equal to the conditional mean. If $p>p_{*}$, the coefficients of degrees greater than $p_{*}$ must be zero. Therefore, if $M_{p_{*}}^{0}$ can be estimated, the most parsimonious polynomial model can be estimated, and the sequential testing procedure described above is designed to estimate $p_{*}$. The WELM testing procedure has the GCR property [10], and the sequential testing procedure starts model testing from the smallest model to larger ones. Therefore, if the lower-degree polynomial model is misspecified for the conditional mean, it will be consistently rejected by the WELM test statistic, so that we can expect to estimate the most parsimonious correct model using the sequential testing procedure. From this result, we obtain the following corollary.

**Corollary** **1.** Given Assumption 1, if Assumptions 2–4 hold for each $p\in P:=\{1,2,\ldots ,\bar{p}\}$ and $p_{*}\in P$, for any ϵ>0, $\lim_{n\to \infty }P(|{\hat{p}}_{n}\left(\alpha \right)-p_{*}\left|>\epsilon \right)=\alpha$, where ${\hat{p}}_{n}\left(\alpha \right)$ is the polynomial degree estimator obtained by applying the WELM test statistics to the sequential testing procedure with the level of significance **α**.

Corollary 1 implies that the degree estimator ${\hat{p}}_{n}\left(\alpha \right)$ has a consistent estimation error equal to the level of significance α; hence, if this estimation error is not removed from the above procedure, the degree estimator is not consistent for $p_{*}$.

Further, the significance level α is selected by the researcher. We can let α be dependent on the sample size *n*, so that, if $\alpha_{n}\to 0$ as n→∞, the degree estimation error can be allowed to converge to zero, leading to a consistent estimator. We contain this result in the following theorem.

**Theorem** **1.** Given Assumption 1, if Assumptions 2 and 3 hold for each $p\in P:=\{1,2,\ldots ,\bar{p}\}$, $p_{*}\in P$, and $\alpha_{n}=1-C\left(c_{n}\right)$ such that for some δ∈(0,1), $c_{n}=O\left(n^{\delta }\right)$, then for any ϵ>0, $\lim_{n\to \infty }P(|{\hat{p}}_{n}\left(\alpha_{n}\right)-p_{*}\left|>\epsilon \right)=0$, where C(·) is the chi-squared distribution function with one degree of freedom.

The results in Corollary 1 and Theorem 1 correspond to the results using the sequential testing procedure in the literature. Hosoya [20] examines the sequential testing procedure for a set of models nested by larger models using the likelihood ratio test statistic, so that the likelihood ratio test statistics can be sequentially applied using the chi-squared null limit distributions. Nevertheless, the models assumed by Hosoya [20] do not have the identification problem that we examine herein. Theorem 2 of Cho and Phillips [17] also provides a result analogous to Theorem 1 of the current study, but their conditions are more relaxed in the following senses. First, they apply the QLR test statistic for their sequential testing problem, which compares the mean square errors obtained from the null and alternative models such that the alternative model is constructed by letting $\Psi (\delta x_{t}=x_{t}^{\delta })$. They show that a multifold identification problem exists under the null that the conditional mean is correctly specified by the polynomial model. Therefore, their QLR test statistic weakly converges to a functional of a Gaussian stochastic process. Consequently, the null limit distribution of their test statistic does not follow a chi-squared distribution. The null limit distribution is obtained using the weighted bootstrapping proposed by Hansen [19], making its application inconvenient. Second, the particular form of power transformation for $\Psi_{t}\left(\cdot \right)$ restricts their applications. If $x_{t}$ is negatively valued, it may not be properly defined. Note that $x_{t}^{\delta }=\exp\left(\delta \log\left(x_{t}\right)\right)$, which is defined only when $x_{t}>0$, so that the application of their methodology is restrictive if $x_{t}$ can be negatively valued. Finally, the level of significance $\alpha_{n}$ is assumed to slowly converge to zero relative to the convergence rate herein. They require that $\log(\alpha_{n})/n\to 0$ in addition to $\alpha_{n}\to 0$, whereas the latter is only assumed in Theorem 1. This requirement is imposed mainly because the null limit distribution of the QLR test statistic is characterized by the maximum of the squared Gaussian process. The tail distribution of the maximum is approximated by associating it with that from the squared fractional Brownian motion using the Slepian inequality. $\log(\alpha_{n})/n\to 0$ is required to yield a sequence of critical values uniformly dominated by those from the squared fractional Brownian motion. On the contrary, our sequential testing procedure does not need to satisfy this additional condition.

## 4. Results: Monte Carlo Simulations

In this section, we illustrate the sequential WELM testing procedure by conducting Monte Carlo simulations using stationary time-series observations.

### 4.1. Linear Function and Sequential Testing Procedure

Without loss of generality, we first suppose the following dynamic and stationary time-series DGP:

$$y_{t}=\alpha_{0*}+\alpha_{1*}x_{t}+\eta_{*}y_{t-1}+\epsilon_{t},$$

where $x_{t}=\phi_{*}x_{t-1}+u_{t}$, ($\epsilon_{t},u_{t})∼$ IID $N(0,\sigma_{*}^{2}I_{2})$, $y_{0}∼N\left(0,\sigma_{y}^{2}\right)$, $x_{0}∼$ IID $N(0,\sigma_{x}^{2})$, and t=1,2,…,n such that $|\phi_{*}|<1$ and $\eta_{*}|<1$. The last two inequality conditions are imposed for the stationarity of the data.

Given this DGP condition, we let our model be constructed by polynomial models. We first consider a linear model as the first-degree polynomial model. For this purpose, we let the explanatory variable vector $x_{t}\left(p\right)$ be simply $x_{t}$, so that p=1, and we also let $d_{t}$ be the lagged dependent variable $y_{t-1}$. Therefore, if we let $\theta ={\left(\alpha_{1},\alpha_{2},\eta \right)}^{\prime }$, the null model $M_{1}^{0}$ becomes {Φ(·,):θ∈Θ}, where $\Phi \left(X_{t},\theta \right):=\alpha_{0}+\alpha_{1}x_{t}+\eta y_{t-1}$. For our alternative model, we let the exponential function be Ψ(·), so that

$$M_{1}:=\left\{f\left(\cdot ;\theta ,\lambda ,\delta \right):\left(\theta ,\lambda ,\delta \right)\in \Theta \times \Lambda \times \Delta \right\}\;\;\;\;\mathrm{and}$$

$$f\left(X_{t};\theta ,\lambda ,\delta \right):=\alpha_{0}+\alpha_{1}x_{t}+\eta y_{t-1}+\lambda \exp\left(\delta x_{t}\right)$$

such that $\Lambda :=[-\bar{\lambda },\bar{\lambda }]$ and $\Delta :=[\underline{\delta },\bar{\delta }]$. Next, we compute the WELM test statistic ${\hat{W}}_{n,m}$ by first approximating ${\bar{\Psi }}_{m,t}:=\int_{\Delta }\Psi \left(X_{t}^{\prime }\delta \right)dQ\left(\delta \right)$ using ${\bar{\Psi }}_{m,t}:=m^{-1}\sum_{i=1}^{m}\exp\left(\delta x_{t}\right)$ and next by letting $w_{t}\left(1\right):={\left[1,x_{t},y_{t-1}\right]}^{\prime }$, where we suppose that Q is a probability measure uniformly distributed on $\Delta =\left[\underline{\delta },\bar{\delta }\right]$. This linear model is correctly specified for the DGP. Therefore, we should expect that the WELM test statistic rejects this model α×100% asymptotically when the level of significance is α.

Next, we extend the model scope to higher-degree polynomial models. For this purpose, we further let $x_{t}\left(p\right):=\left(1,x_{t},x_{t}^{2},\ldots ,x_{t}^{p}\right)$ to specify the following null and alternative models:

$$M_{p}^{0}=\left\{\alpha_{0}+\alpha_{1}x_{t}+\alpha_{2}x_{t}^{2}+\cdots +\alpha_{p}x_{t}^{p}+\eta y_{t-1}:\theta :=\left(\alpha_{0},\ldots ,\alpha_{p},\eta \right)\in \Theta \left(p\right)\right\},\;\;\;\mathrm{and}$$

$$M_{p}=\left\{\alpha_{0}+\alpha_{1}x_{t}+\alpha_{2}x_{t}^{2}+\cdots +\alpha_{p}x_{t}^{p}+\eta y_{t-1}+\lambda \exp\left(\delta x_{t}\right):\theta :=\left(\alpha_{0},\ldots ,\alpha_{p},\eta \right)\in \Theta \left(p\right),\lambda \in \Lambda ,\delta \in \Delta \right\}.$$

Given this, we further let $w_{t}\left(p\right):={\left[1,x_{t},x_{t}^{2},\ldots ,x_{t}^{p},y_{t-1}\right]}^{\prime }$ to compute the WELM test statistic to test the *p*-th-degree polynomial model. If p=1, the WELM test statistic is the same as that obtained using the linear model. For p=2,3 and $\bar{p}=4$, the null models $M_{p}^{0}$ are correctly specified, so that the WELM test statistics are also expected to reject the null model α×100% asymptotically. Through this, we construct the following sets of alternative and null models:

$$M\left(4\right):=\left\{M_{p}:p=1,2,\ldots ,4\right\}\;\;\;\mathrm{and}\;\;\;M^{0}\left(4\right):=\left\{M_{p}^{0}:p=1,2,\ldots ,4\right\}$$

to apply the sequential testing procedure.

For this DGP and the models, we conduct simulations and report the simulation results in Table 1, which are obtained by applying the sequential testing procedure. We generate data by letting $\left(\alpha_{1*},\eta_{*},\phi_{*},\sigma_{*}^{2}\right)=\left(0.5,0.5,0.5,1.0\right)$ and also let the levels of significance α be 10%, 5%, and 1%. Given these simulation environments, we examine the empirical rejection rates of the WELM test statistic for n=50, 100, 200, 500, 1000, 2000, and 5000. We also let m=5000, and the total number of experiments is 5000. In the Supplementary Materials, we provide the URL address containing this simulation code made in R language.

The simulation results can be summarized as follows. First, the sequential testing procedure stops mostly at the first step, which implies that the sequential WELM test identifies the correct degree of the unknown polynomial function correctly. More specifically, as the sample size *n* increases, the WELM test statistic detects the linear model as the correct model approximately (1−α)×100%. This aspect is observed irrespective of the sample size, so that we can expect that the WELM test statistic controls the type-I error precisely; hence, the most parsimonious correct model can be efficiently estimated. Second, even when the sequential testing procedure estimates the models whose polynomial degrees are greater than unity, most of the selected models are quadratic models. This implies that the sequential testing procedure has a strong tendency to select the next most parsimonious model for the conditional mean function. As a result, selected models are mostly linear or quadratic functions. Third, as the level of significance α decreases, more precise estimation results are delivered from the experiments. However, this result in another way implies that the estimation error cannot be eliminated altogether as long as the level of significance is fixed.

We therefore conduct another simulation by letting the level of significance be dependent upon the sample size. Specifically, we let the level of significance $\alpha_{n}$ be $n^{-1/2}$, $n^{-1}$, $n^{-3/2}$, and $n^{-2}$. For these levels of significance, $\alpha_{n}$ reduces to zero as *n* increases, so that the sequential testing procedure is expected to eliminate the estimation error asymptotically. Among the levels of significance, $n^{-2}$ approaches zero more quickly than the other levels of significance. Table 2 reports the simulation results obtained from 5000 experiments. The figures in the first panel denote ${\hat{P}}_{n}\left(\alpha_{n}\right):=r^{-1}\sum_{i=1}^{r}I\left({\hat{p}}_{n,i}=1\right)$, where *r* denotes the total number of experiments set to be 5000 and ${\hat{p}}_{n,i}$ denotes the degree estimated by the sequential testing procedure from the *i*-th experiment when the level of significance is $\alpha_{n}$. Here, I(·) denotes the indicator function. For each plan for the level of significance $\alpha_{n}$, ${\hat{P}}_{n}\left(\alpha_{n}\right)$ estimates the empirical probability for the estimated degree using the sequential testing procedure to be equal to 100%. The other figures in parentheses denote the hypothetical proportion measured by $(1-\alpha_{n})\times 100$%. As $\alpha_{n}$ reduces to zero more quickly, the hypothetical proportion more quickly arrives at 100%. In addition to the sequential testing procedure, we compare these estimation results with standard information criterion-based estimations using the Akaike information criterion (AIC), Bayesian information criterion (BIC), and small-sample corrected AIC (AICc). These information criteria are applied to the null models $M_{p}^{0}$ with p=1,2,3,4 and we compute the proportions measured by ${\tilde{P}}_{n}:=r^{-1}\sum_{i=1}^{r}I\left({\tilde{p}}_{n,i}=1\right)$, where ${\tilde{p}}_{n,i}$ denotes the degree selected by the information criterion. The figures in the second panel report the proportions estimated by the information criteria. Finally, we apply the same information criteria to the alternative models $M_{p}$ with p=1,2,3,4 and report the estimated proportions in the third panel, which are obtained using the same methodology. We distinguish them from the earlier information criteria by attaching “${}^{\prime }$” to AIC, BIC, and AICc, so that AIC${}^{\prime }$, BIC${}^{\prime }$, and AICc${}^{\prime }$ denote the information criteria applied to the alternative models.

The simulation results in Table 2 can be summarized as follows. First, for every significance level $\alpha_{n}$, the distance between ${\hat{P}}_{n}\left(\alpha_{n}\right)$ and $\left(1-\alpha_{n}\right)$ approaches zero as the sample size *n* increases. This suggests that the first-degree polynomial model is successfully estimated using the sequential estimation procedure. Second, the distance between ${\hat{P}}_{n}\left(\alpha_{n}\right)$ and $\left(1-\alpha_{n}\right)$ is closest to zero when the plan for the level of significance is set to $\alpha_{n}=n^{-2}$. This implies that the sequential testing procedure can estimate the first-degree polynomial model more precisely than xxxx by letting the level of significance converge to zero more quickly. Third, as the second panel shows, the BIC converges to 100% with the increase in sample size, whereas the AIC and AICc are not as fast as the BIC. Fourth, as the third panel shows, the BIC${}^{\prime }$ performs similarly to the BIC, whereas the AIC${}^{\prime }$ and AICc${}^{\prime }$ perform a little worse than the AIC and AICc, respectively. Finally, when comparing the BIC (or the BIC${}^{\prime }$) with the sequential WELM testing procedure, the performance of the information criteria is inferior to those of the sequential testing procedure when $\alpha_{n}$ converges to zero quickly. Specifically, if we let $\alpha_{n}$ be $n^{-3/2}$ or $n^{-2}$, the performance of the sequential testing procedure is better than that obtained by the BIC for all sample sizes. By contrast, if we let $\alpha_{n}$ be $n^{-1/2}$, the performance of the BIC is superior to the sequential testing procedure for all sample sizes. In the middle, if $\alpha_{n}$ reduces to zero at a moderate rate, namely $n^{-1}$, the performance of the sequential testing procedure is dependent upon the sample size *n*. That is, if *n* is relatively small, the sequential testing procedure performs better than the BIC; however, if *n* is relatively large, the BIC performs better than the sequential testing procedure. This aspect implies that letting the level of significance converge to zero as quickly as possible can produce the best estimation result if the first-degree polynomial model is a correct model.

### 4.2. Quadratic Function and Sequential Testing Procedure

We extend the earlier simulation by conducting another simulation. We examine a different DGP. Specifically, we suppose that data are generated by $y_{t}=\alpha_{1*}x_{t}+\alpha_{2*}x_{t}^{2}+\eta_{*}y_{t-1}+\epsilon_{t}$, where $x_{t}=\phi_{*}x_{t-1}+u_{t}$, $(x_{0},y_{0})∼$ IID $N(0,I_{2})$, ($\epsilon_{t},u_{t})∼$ IID $N(0,\sigma_{*}^{2}I_{2})$, and $\left(\alpha_{1*},\alpha_{2*},\eta_{*},\phi_{*},\sigma_{*}^{2}\right)=\left(0.5,0.5,0.5,0.5,1.0\right)$. Therefore, the first-degree polynomial model is now incorrectly specified, whereas the second-, third-, and fourth-degree polynomial models are correctly specified. Hence, the desired sequential testing procedure should estimate the second-degree polynomial model as the most parsimonious and correctly specified model. We attempt this second simulation to verify whether the lessons we could have obtained from the simulations in Section 4.1 are still valid for other DGPs.

As before, we first conduct the simulations by fixing the levels of significance and next by letting them depend on the sample size. Table 3 and Table 4, respectively, report the simulation results for the first and second cases obtained in the same simulation environments as for Table 1 and Table 2.

We can summarize the simulation results as follows. First, Table 3 shows that the proportion of the linear model selected by the sequential testing procedure decreases to zero as the sample size *n* increases. For each level of significance, 10%, 5%, and 1%, the first-degree polynomial model is selected less and less as *n* increases. This aspect implies that the WELM test statistic has a consistent power to reject the misspecified model. Second, as shown in Table 3, the second-degree polynomial model is asymptotically selected (1−α)×100%, and this implies that the WELM test statistic controls the type-I error efficiently. Hence, the most parsimonious and correctly specified model can be consistently selected by the sequential testing procedure. Third, as before, the estimation error incurred by the sequential testing procedure cannot be removed altogether as long as the level of significance is fixed irrespective of the sample size. Fourth, Table 4 reports the proportions of the polynomial degrees estimated by the sequential WELM testing procedure with the significance levels dependent on the sample size, and the information criteria. As we can see, for $\alpha_{n}=n^{-1}$, $\alpha_{n}=n^{-6/4}$, and $\alpha_{n}=n^{-2}$, the distance between ${\hat{P}}_{n}\left(\alpha_{n}\right)$ and $\left(1-\alpha_{n}\right)$ approaches zero with an increase in sample size. Fifth, if the sample size is relatively small, slowly converging levels of significance estimate the correct degree better than quickly converging levels. For example, if n=50, letting $\alpha_{n}=n^{-1/2}$ produces higher proportions than that obtained by letting $\alpha_{n}$ be $n^{-2}$. Nevertheless, as the sample size increases, they show different estimation patterns. For $\alpha_{n}=n^{-1/2}$, the proportion converges to 100% slowly, whereas for $\alpha_{n}=n^{-2}$, it converges to 100% quickly, implying that the plans for the level of significance have to be carefully chosen to apply them to the sequential testing procedure. If a relatively large sample data set is examined, the correct degree of the polynomial model can be better estimated by letting the level of significance converge to zero quickly. On the contrary, if the sample size is small, a level of significance converging to zero relatively slowly should be chosen. Sixth, we also compare the performances of the information criteria and observe that the BIC overall performs better than the AIC and AICc, and the same thing holds among the AIC${}^{\prime }$, BIC${}^{\prime }$, and AICc${}^{\prime }$. Further, the BIC always provides better estimates than the BIC${}^{\prime }$. Finally, we compare the simulation results using the sequential testing procedure with the BIC. If the sample size is small, the BIC always dominates all the estimation results from using the sequential testing procedures; however, if the sample size is sufficiently large, say more than 2000, the sequential testing procedure with a level of significance converging to zero quickly provides better estimates than the BIC. This simulation result is different from what we observed in Section 4.1. The sequential testing procedure does not always perform better than the BIC. If the polynomial function has a lower degree in the DGP, the sequential testing procedure may perform better than the information criterion. In particular, if the sample size is sufficiently large, the use of the sequential testing procedure appears more amenable.

### 4.3. Misspecified Models and Sequential Testing Procedure

As our final simulation, we now suppose that none of the models are correctly specified by supposing that $y_{t}=\pi_{*}\cos\left(y_{t-1}\right)+\epsilon_{t}$, where $y_{0}∼N\left(0,\sigma_{y_{0}}^{2}\right)$ and $u_{t}∼$ IID $N(0,\sigma_{u}^{2})$. Here, we let $\left(\pi_{*},\sigma_{y_{0}}^{2},\sigma_{u}^{2}\right)=\left(1.0,1.0,1.0\right)$. We apply the same models as before and select the best model using the sequential testing procedure. Note that the cos(·) function is expressed as an infinite-degree polynomial function by Taylor’s expansion, so that the fourth-degree polynomial model cannot be correctly specified for this DGP. This implies that the sequential testing procedure is expected to estimate a degree greater than 4. Our primary interest in this simulation is in investigating how the earlier finite sample properties of the sequential testing procedure are modified by this new DGP condition. As the model conditions and simulation environments are the same as before, we do not iterate.

Table 5 and Table 6 report the simulation results. Table 5 is obtained by fixing the levels of significance and Table 6 is obtained by letting the levels of significance depend on the sample size. The simulation results are summarized as follows. First, as the sample size *n* increases, the empirical rejection rates also increase for each degree p=1,2, and 3 and the sequential testing procedure concludes that the polynomial degree is greater than or equal to 4 for most experiments. For example, if n=2000, the sums of the proportions of p=1,2,3 are only 0.58%, 4.82%, and 11.55% for the 10%, 5%, and 1% significance levels, respectively, and they further decrease as *n* increases to 5000. This result indicates that the power of the sequential WELM testing procedure performs well if the sample size is sufficiently large. Second, when an incorrect model is selected, the quadratic model is overall selected more often than the linear or cubic models. That is, the second-degree polynomial model is preferred to the first- and third-degree polynomial models. This is mainly because the cosine function is an even function around zero, so that the quadratic function may better approximate the cosine function when the sample size is not sufficiently large. Third, we now let the levels of significance depend on the sample size and examine the simulation results in Table 6.

As we can see, the distance between ${\hat{P}}_{n}\left(\alpha_{n}\right)$ and $\left(1-\alpha_{n}\right)$ reduces with a rise in sample size. Although the distance is not as close to zero as in Table 2 and Table 4, the distance reduces. Further, if *n* is small, slowly converging levels of significance provide better estimates than quickly converging plans. Nevertheless, as *n* increases, the proportions converge to 100% more quickly when we let $\alpha_{n}$ be $n^{-2}$ than when we let $\alpha_{n}$ be $n^{-1/2}$. Hence, if the data set has a large sample size, the level of significance converging to zero relatively quickly should be chosen. This is the same observation as in Section 4.2. Moreover, we now compare the performances of the information criteria and observe that the AIC overall performs better than the BIC and AICc, and the same thing holds among the AIC${}^{\prime }$, BIC${}^{\prime }$, and AICc${}^{\prime }$. In addition, the AIC always provides better estimates than the AIC${}^{\prime }$. Finally, we compare the simulation results using the sequential testing procedure with the AIC. The AIC always dominates all the estimations from using the sequential testing procedures. This simulation result implies that the BIC is not always the best performing information criterion and the sequential testing procedure can dominate the BIC even when the sample size is small. Furthermore, if all the considered models are misspecified, it is difficult to draw regular patterns among the sequential testing procedure and information criteria.

## 5. Conclusions

We applied the Wald test statistic assisted by the ELM to test the correct model assumption and estimate a close approximation of the conditional mean. When testing for the model misspecification of the conditional mean, omnibus test statistics typically weakly converge to a Gaussian stochastic process under the null hypothesis that the model is correctly specified. This aspect makes their applications inconvenient. We defined the Wald test statistic using the functional regression and applied the ELM to compute the test statistic efficiently (i.e., WELM), following Cho and White [15]. The WELM test statistic is GCR and follows a chi-squared distribution under the null. We further applied the WELM test statistic to a sequential testing procedure to search for an approximate conditional expectation and conduct extensive Monte Carlo experiments to evaluate its performance. Using simulation, we verified that, if the candidate polynomial models are correctly specified, the sequential WELM testing procedure estimates the most parsimonious and correct model consistently. Further, it consistently rejects all the candidate models if none of the polynomial models are correctly specified. We further compared the performance of standard information criteria, such as the BIC and AIC, as well as its small-sample adjusted version. From this comparison, we find that the model estimation using the sequential testing procedure has competitive power in estimating the most parsimonious and correct model.

## Figures and Tables

**Table 1:** Estimated polynomial degrees using the sequential Wald extreme learning machine (WELM) testing procedure (in percent). Number of replications: 5000. This table reports the proportion of estimated polynomial degrees using the sequential WELM testing procedure. DGP: $y_{t}=\alpha_{1*}x_{t}+\eta_{*}y_{t-1}+\epsilon_{t}$, where $x_{t}=\phi_{*}x_{t-1}+u_{t}$, $(x_{0},y_{0})∼$ IID $N(0,I_{2})$, ($\epsilon_{t},u_{t})∼$ IID $N(0,\sigma_{*}^{2}I_{2})$, $\delta_{i}∼$ IID U(0,1), and $\left(\alpha_{1*},\eta_{*},\phi_{*},\sigma_{*}^{2}\right)=\left(0.5,0.5,0.5,1.0\right)$. Here, the given hypotheses are provided as follows: $H_{0}^{\left(1\right)}:E\left[y_{t}|x_{t},y_{t-1}\right]=\theta_{0*}+\theta_{1*}x_{t}+\theta_{y*}y_{t-1}$; $H_{0}^{\left(2\right)}:E\left[y_{t}|x_{t},y_{t-1}\right]=\theta_{0*}+\theta_{1*}x_{t}+\theta_{2*}x_{t}^{2}+\theta_{y*}y_{t-1}$; $H_{0}^{\left(3\right)}:E\left[y_{t}|y_{t-1}\right]=\theta_{0*}+\theta_{1*}x_{t}+\theta_{2*}x_{t}^{2}+\theta_{3*}x_{t}^{3}+\theta_{y*}y_{t-1}$; and $H_{0}^{\left(4\right)}:E\left[y_{t}|x_{t}\right]=\theta_{0*}+\theta_{1*}x_{t}+\theta_{2*}x_{t}^{2}+\theta_{3*}x_{t}^{3}+\theta_{4*}x_{t}^{4}+\theta_{y*}y_{t-1}$. We further let $\Psi \left(x_{t}\delta \right)=\exp\left(x_{t}\delta \right)$ to compute the WELM test statistic.

Nominal Level (%)	p∖n	50	100	200	500	1000	2000	5000
10%	1	89.88	89.82	89.48	90.36	89.94	89.92	89.70
	2	9.02	9.04	8.78	8.10	8.12	7.72	7.90
	3	0.94	1.02	1.66	1.46	1.66	2.10	2.00
	≥4	0.16	0.12	0.08	0.08	0.28	0.26	0.40
5%	1	96.12	95.92	96.44	95.5	94.4	95.24	95.18
	2	3.6	3.84	3.38	4.1	4.98	4.18	4.16
	3	0.28	0.22	0.18	0.34	0.56	0.5	0.54
	≥4	0	0.02	0	0.06	0.06	0.08	0.12
1%	1	99.69	99.5	99.44	99.41	99.3	99.25	99.29
	2	0.31	0.5	0.56	0.59	0.7	0.75	0.71
	3	0	0	0	0	0	0	0
	≥4	0	0	0	0	0	0	0

**Table 2:** Proportion of sequentially estimated polynomial degrees using the sequential WELM testing procedure (in percent). Number of replications: 5000. This table reports the percentages of the correctly estimated polynomial degree using the sequential WELM testing procedure and the information criteria. The figures in the first panel denote ${\hat{P}}_{n}\left(\alpha_{n}\right)\times 100$, and those in the second and third panels are ${\tilde{P}}_{n}\times 100$. In addition, the figures in parentheses denote $(1-\alpha_{n})\times 100$, where we let ${\hat{P}}_{n}\left(\alpha_{n}\right):=r^{-1}\sum_{i=1}^{r}I\left({\hat{p}}_{n,i}=p_{*}\right)$. *r* is the number of iterations, ${\hat{p}}_{n,i}$ denotes the degree estimator obtained from the sequential testing procedure for the *i*-th simulation, and I(·) is the indicator function. Similarly, ${\tilde{P}}_{n}:=r^{-1}\sum_{i=1}^{r}I\left({\tilde{p}}_{n,i}=p_{*}\right)$, where ${\tilde{p}}_{n,i}$ is the degree estimator obtained by the information criteria. MODEL: $M_{p}:=\left\{x_{t}{\left(p\right)}^{\prime }\alpha \left(p\right)+\eta y_{t-1}+\Psi \left(\delta x_{t}\right)\right\}$, where p=1,2,3,4. The Akaike information criterion (AIC), Bayesian information criterion (BIC), and small-sample corrected AIC (AICc) are the information criteria applied to $M_{p}^{0}:=\left\{x_{t}{\left(p\right)}^{\prime }\alpha \left(p\right)+\eta y_{t-1}\right\}$, and the AIC${}^{\prime }$, BIC${}^{\prime }$, and AICc${}^{\prime }$ are those applied to $M_{p}$, where p=1,2,3,4. DGP: $y_{t}=\alpha_{1*}x_{t}+\eta_{*}y_{t-1}+\epsilon_{t}$, where $x_{t}=\phi_{*}x_{t-1}+u_{t}$, $(x_{0},y_{0})∼$ IID $N(0,I_{2})$, ($\epsilon_{t},u_{t})∼$ IID $N(0,\sigma_{*}^{2}I_{2})$, $\delta_{i}∼$ IID U(0,1), and $\left(\alpha_{1*},\eta_{*},\phi_{*},\sigma_{*}^{2}\right)=\left(0.5,0.5,0.5,1.0\right)$.

Methods∖n	50	100	200	500	1000	2000	5000
Seqn. Estmtn.	84.06	85.48	85.28	84.84	85.76	86.18	86.82
with $\alpha_{n}=n^{-1/2}$	(85.86)	(90.00)	(92.93)	(95.53)	(96.83)	(97.76)	(98.59)
Seqn. Estmtn.	98.90	98.80	98.40	98.30	98.48	98.06	98.22
with $\alpha_{n}=n^{-1}$	(98.00)	(99.00)	(99.50)	(99.80)	(99.90)	(99.95)	(99.98)
Seqn. Estmtn.	100.0	99.90	99.88	99.90	98.78	99.84	99.72
with $\alpha_{n}=n^{-3/2}$	(99.71)	(99.90)	(99.96)	(99.99)	(100.0)	(100.0)	(100.0)
Seqn. Estmtn.	100.0	100.0	99.98	99.96	100.0	99.98	100.0
with $\alpha_{n}=n^{-2}$	(99.96)	(99.99)	(100.0)	(100.0)	(100.0)	(100.0)	(100.0)
AIC	75.40	77.74	77.64	76.74	78.04	78.8	78.16
BIC	92.86	95.48	97.24	98.34	98.92	99.34	99.68
AICc	81.34	80.56	78.68	77.38	78.30	78.86	78.24
AIC${}^{\prime }$	70.64	73.94	73.94	75.76	75.54	76.14	76.22
BIC${}^{\prime }$	92.12	95.80	97.74	98.56	99.02	99.38	99.68
AICc${}^{\prime }$	78.84	77.50	76.30	76.34	75.94	76.34	76.32

**Table 3:** Estimated polynomial degrees using the sequential WELM testing procedure (in percent). Number of replications: 5000. This table reports the proportion of estimated polynomial degrees using the sequential WELM testing procedure. DGP: $y_{t}=\alpha_{1*}x_{t}+\alpha_{2*}x_{t}^{2}+\eta_{*}y_{t-1}+\epsilon_{t}$, where $x_{t}=\phi_{*}x_{t-1}+u_{t}$, $(x_{0},y_{0})∼$ IID $N(0,I_{2})$, ($\epsilon_{t},u_{t})∼$ IID $N(0,\sigma_{*}^{2}I_{2})$, $\delta_{i}∼$ IID U(0,1), and $\left(\alpha_{1*},\alpha_{2*},\eta_{*},\phi_{*},\sigma_{*}^{2}\right)=\left(0.5,0.5,0.5,0.5,1.0\right)$. Here, the hypotheses are provided as follows: $H_{0}^{\left(1\right)}:E\left[y_{t}|x_{t},y_{t-1}\right]=\theta_{0*}+\theta_{1*}x_{t}+\theta_{y*}y_{t-1}$; $H_{0}^{\left(2\right)}:E\left[y_{t}|x_{t},y_{t-1}\right]=\theta_{0*}+\theta_{1*}x_{t}+\theta_{2*}x_{t}^{2}+\theta_{y*}y_{t-1}$; $H_{0}^{\left(3\right)}:E\left[y_{t}|y_{t-1}\right]=\theta_{0*}+\theta_{1*}x_{t}+\theta_{2*}x_{t}^{2}+\theta_{3*}x_{t}^{3}+\theta_{y*}y_{t-1}$; and $H_{0}^{\left(4\right)}:E\left[y_{t}|x_{t}\right]=\theta_{0*}+\theta_{1*}x_{t}+\theta_{2*}x_{t}^{2}+\theta_{3*}x_{t}^{3}+\theta_{4*}x_{t}^{4}+\theta_{y*}y_{t-1}$. We further let $\Psi \left(x_{t}\delta \right)=\exp\left(x_{t}\delta \right)$ to compute the WELM test statistic.

Nominal Level (%)	p∖n	50	100	200	500	1000	2000	5000
10%	1	7.56	2.18	0.78	0.16	0.06	0.00	0.00
	2	81.30	87.14	89.10	89.40	90.48	89.98	90.20
	3	9.56	9.22	8.98	8.94	8.18	8.56	8.08
	≥4	1.58	1.46	1.14	1.50	1.28	1.46	1.72
5%	1	21.36	7.38	3.32	0.56	0.16	0.00	0.00
	2	74.58	88.28	91.72	95.02	96.02	95.92	95.30
	3	3.80	4.12	4.66	4.10	3.40	3.64	4.28
	≥4	0.26	0.22	0.30	0.32	0.42	0.44	0.42
1%	1	61.34	28.62	12.46	2.86	0.96	0.14	0.02
	2	38.32	70.94	86.90	96.74	98.36	98.96	99.34
	3	0.34	0.44	0.60	0.40	0.66	0.88	0.62
	≥4	0.00	0.00	0.04	0.00	0.02	0.02	0.02

**Table 4:** Proportion of sequentially estimated polynomial degrees using the sequential WELM testing procedure (in percent). Number of replications: 5000. This table reports the percentages of the correctly estimated polynomial degree using the sequential WELM testing procedure and the information criteria. The figures in the first panel denote ${\hat{P}}_{n}\left(\alpha_{n}\right)\times 100$ and those in the second and third panels are ${\tilde{P}}_{n}\times 100$. In addition, the figures in parentheses denote $(1-\alpha_{n})\times 100$, where we let ${\hat{P}}_{n}\left(\alpha_{n}\right):=r^{-1}\sum_{i=1}^{r}I\left({\hat{p}}_{n,i}=p_{*}\right)$; *r* is the number of iterations. ${\hat{p}}_{n,i}$ denotes the degree estimator obtained using the sequential testing procedure for the *i*-th simulation and I(·) is the indicator function. Similarly, ${\tilde{P}}_{n}:=r^{-1}\sum_{i=1}^{r}I\left({\tilde{p}}_{n,i}=p_{*}\right)$, where ${\tilde{p}}_{n,i}$ is the degree estimator obtained by the information criteria. MODEL: $M_{p}:=\left\{x_{t}{\left(p\right)}^{\prime }\alpha \left(p\right)+\eta y_{t-1}+\Psi \left(\delta x_{t}\right)\right\}$, where p=1,2,3,4. The AIC, BIC, and AICc are the information criteria applied to $M_{p}^{0}:=\left\{x_{t}{\left(p\right)}^{\prime }\alpha \left(p\right)+\eta y_{t-1}\right\}$, and the AIC${}^{\prime }$, BIC${}^{\prime }$, and AICc${}^{\prime }$ are those applied to $M_{p}$, where p=1,2,3,4. DGP: $y_{t}=\alpha_{1*}x_{t}+\alpha_{2*}x_{t}^{2}+\eta_{*}y_{t-1}+\epsilon_{t}$, where $x_{t}=\phi_{*}x_{t-1}+u_{t}$, $(x_{0},y_{0})∼$ IID $N(0,I_{2})$, ($\epsilon_{t},u_{t})∼$ IID $N(0,\sigma_{*}^{2}I_{2})$, $\delta_{i}∼$ IID U(0,1), and $\left(\alpha_{1*},\alpha_{2*},\eta_{*},\phi_{*},\sigma_{*}^{2}\right)=\left(0.5,0.5,0.5,0.5,1.0\right)$.

Methods∖n	50	100	200	500	1000	2000	5000
Seqn. Estmtn.	80.18	83.12	83.34	85.00	86.28	85.80	85.62
with $\alpha_{n}=n^{-1/2}$	(85.86)	(90.00)	(92.93)	(95.53)	(96.84)	(97.76)	(98.59)
Seqn. Estmtn.	53.98	81.48	90.78	96.52	98.00	98.10	98.44
with $\alpha_{n}=n^{-1}$	(94.68)	(96.84)	(98.12)	(99.05)	(99.44)	(99.67)	(99.83)
Seqn. Estmtn.	15.70	49.46	76.74	93.28	97.82	99.48	99.84
with $\alpha_{n}=n^{-3/2}$	(99.71)	(99.90)	(99.96)	(99.99)	(100.0)	(100.0)	(100.0)
Seqn. Estmtn.	2.48	20.66	56.92	86.98	95.88	98.98	99.90
with $\alpha_{n}=n^{-2}$	(99.96)	(99.99)	(100.0)	(100.0)	(100.0)	(100.0)	(100.0)
AIC	81.08	83.92	83.26	84.66	84.08	83.94	83.86
BIC	92.22	96.50	97.44	98.66	98.92	99.50	99.64
AICc	85.42	85.68	84.22	85.16	84.28	84.08	83.90
AIC${}^{\prime }$	64.90	76.28	78.16	78.80	78.74	78.64	77.78
BIC${}^{\prime }$	71.44	93.54	97.56	98.70	99.12	99.22	99.74
AICc${}^{\prime }$	69.60	80.10	79.80	79.48	79.04	78.86	77.86

**Table 5:** Estimated polynomial degrees using the sequential WELM testing procedure (in percent). Number of replications: 5000. This table reports the proportion of estimated polynomial degrees using the sequential WELM testing procedure. DGP: $y_{t}=\pi_{*}\cos\left(y_{t-1}\right)+\epsilon_{t}$, where $y_{0}∼N\left(0,\sigma_{y_{0}}^{2}\right)$, $\delta_{i}∼$ IID U(−1,1), and $u_{t}∼$ IID $N(0,\sigma_{u}^{2})$. Here, we let $\left(\pi_{*},\sigma_{y_{0}}^{2},\sigma_{u}^{2}\right)=\left(1.0,1.0,1.0\right)$. The hypotheses are provided as follows: $H_{0}^{\left(1\right)}:E\left[y_{t}|x_{t},y_{t-1}\right]=\theta_{0*}+\theta_{1*}x_{t}+\theta_{y*}y_{t-1}$; $H_{0}^{\left(2\right)}:E\left[y_{t}|x_{t},y_{t-1}\right]=\theta_{0*}+\theta_{1*}x_{t}+\theta_{2*}x_{t}^{2}+\theta_{y*}y_{t-1}$; $H_{0}^{\left(3\right)}:E\left[y_{t}|y_{t-1}\right]=\theta_{0*}+\theta_{1*}x_{t}+\theta_{2*}x_{t}^{2}+\theta_{3*}x_{t}^{3}+\theta_{y*}y_{t-1}$; and $H_{0}^{\left(4\right)}:E\left[y_{t}|x_{t}\right]=\theta_{0*}+\theta_{1*}x_{t}+\theta_{2*}x_{t}^{2}+\theta_{3*}x_{t}^{3}+\theta_{4*}x_{t}^{4}+\theta_{y*}y_{t-1}$. All these null hypotheses are misspecified for the DGP. We further let $\Psi \left(x_{t}\delta \right)=\exp\left(x_{t}\delta \right)$ to compute the WELM test statistic.

Nominal Level (%)	p∖n	50	100	200	500	1000	2000	5000
10%	1	20.50	5.10	1.10	0.08	0.04	0.00	0.00
	2	56.46	51.04	30.90	6.78	1.94	0.58	0.12
	3	11.24	17.90	18.88	6.08	0.36	0.00	0.00
	≥4	11.8	25.96	49.12	87.06	97.66	99.42	99.88
5%	1	39.82	13.50	3.76	0.86	0.26	0.00	0.00
	2	55.16	71.94	66.90	42.70	19.30	4.82	0.30
	3	4.22	11.30	18.22	11.90	1.76	0.00	0.00
	≥4	0.80	3.17	11.12	44.54	78.68	95.18	99.70
1%	1	71.18	30.31	5.80	0.50	0.04	0.00	0.00
	2	27.98	65.51	78.66	48.81	22.47	9.36	1.91
	3	0.42	2.34	8.76	16.90	9.06	2.19	0.67
	≥4	0.42	1.84	6.78	33.79	68.43	88.45	97.42

**Table 6:** Proportion of sequentially estimated polynomial degrees using the sequential WELM testing procedure (in percent). Number of replications: 5000. This table reports the percentages of the correctly estimated polynomial degree using the sequential WELM testing procedure and the information criteria. The figures in the first panel denote ${\hat{P}}_{n}\left(\alpha_{n}\right)\times 100$ and those in the second and third panels are ${\tilde{P}}_{n}\times 100$. In addition, the figures in parentheses denote $(1-\alpha_{n})\times 100$, where we let ${\hat{P}}_{n}\left(\alpha_{n}\right):=r^{-1}\sum_{i=1}^{r}I\left({\hat{p}}_{n,i}=p_{*}\right)$. *r* is the number of iterations, ${\hat{p}}_{n,i}$ denotes the degree estimator obtained using the sequential testing procedure for the *i*-th simulation, and I(·) is the indicator function. Similarly, ${\tilde{P}}_{n}:=r^{-1}\sum_{i=1}^{r}I\left({\tilde{p}}_{n,i}=p_{*}\right)$, where ${\tilde{p}}_{n,i}$ is the degree estimator obtained by the information criteria. MODEL: $M_{p}:=\left\{x_{t}{\left(p\right)}^{\prime }\alpha \left(p\right)+\eta y_{t-1}+\Psi \left(\delta x_{t}\right)\right\}$, where p=1,2,3,4. The AIC, BIC, and AICc are the information criteria applied to $M_{p}^{0}:=\left\{x_{t}{\left(p\right)}^{\prime }\alpha \left(p\right)+\eta y_{t-1}\right\}$, and the AIC${}^{\prime }$, BIC${}^{\prime }$, and AICc${}^{\prime }$ are those applied to $M_{p}$, where p=1,2,3,4. DGP: $y_{t}=\pi_{*}\cos\left(y_{t-1}\right)+\epsilon_{t}$, where $y_{0}∼N\left(0,\sigma_{y_{0}}^{2}\right)$, $\delta_{i}∼$ IID U(−1,1), $u_{t}∼$ IID $N(0,\sigma_{u}^{2})$, and $\left(\pi_{*},\sigma_{y_{0}}^{2},\sigma_{u}^{2}\right)=\left(1.0,1.0,1.0\right)$.

Methods∖n	50	100	200	500	1000	2000	5000
Seqn. Estmtn.	14.38	27.76	47.30	67.90	61.68	41.80	11.80
with $\alpha_{n}=n^{-1/2}$	(85.86)	(90.00)	(92.93)	(95.53)	(96.84)	(97.76)	(98.59)
Seqn. Estmtn.	4.32	11.36	29.04	63.24	75.84	63.72	31.08
with $\alpha_{n}=n^{-3/4}$	(94.68)	(96.84)	(98.12)	(99.05)	(99.44)	(99.67)	(99.83)
Seqn. Estmtn.	1.04	4.42	14.36	47.30	74.64	78.58	54.68
with $\alpha_{n}=n^{-1}$	(98.00)	(99.00)	(99.50)	(99.80)	(99.90)	(99.95)	(99.98)
Seqn. Estmtn.	0.00	0.00	0.16	2.90	19.50	53.60	81.64
with $\alpha_{n}=n^{-2}$	(99.96)	(99.99)	(100.0)	(100.0)	(100.0)	(100.0)	(100.0)
AIC	19.74	32.94	59.70	94.26	99.74	100.0	100.0
BIC	5.38	9.26	22.08	65.66	96.16	99.98	100.0
AICc	15.12	29.90	58.22	94.14	99.74	100.0	100.0
AIC${}^{\prime }$	8.82	9.64	13.06	26.14	45.72	76.26	98.14
BIC${}^{\prime }$	0.90	0.74	0.88	2.00	5.72	19.92	67.76
AICc${}^{\prime }$	5.34	7.84	11.84	25.50	45.40	76.12	98.12

## Supplementary Materials

The program codes to reproduce the simulation outputs are available online at https://web.yonsei.ac.kr/jinseocho/swelm.htm.

## Appendix A

**Proof** **of Lemma 1.** (*i*) First, Theorem 2 of Cho, Huang, and White [14] implies that

(A1) $$\left[\begin{array}{c} \sqrt{n}\int_{\Delta }\left({\hat{\alpha }}_{n}\left(\delta \right)-\alpha_{*}\left(\delta \right)\right)dQ\left(\delta \right)\\ \sqrt{n}\int_{\Delta }\left({\hat{\beta }}_{n}\left(\delta \right)-\beta_{*}\left(\delta \right)\right)dQ\left(\delta \right) \end{array}\right]\overset{A}{∼}N\left[\left(\begin{array}{c} 0\\ 0 \end{array}\right),\left(\begin{array}{cc} \sigma_{\gamma }^{2} & 0\\ 0 & \sigma_{\xi }^{2}/\sigma_{u}^{2} \end{array}\right)\right],$$

so that

$$\sqrt{n}\int_{\Delta }{\hat{\beta }}_{n}\left(\delta \right)dQ\left(\delta \right)\overset{A}{∼}N\left(0,\sigma_{\xi }^{2}/\sigma_{u}^{2}\right)$$

under $H_{0}^{\prime \prime \prime }$. Therefore,

$$W_{n}:=n\left(\frac{{\hat{\sigma }}_{u,n}^{2}}{{\hat{\sigma }}_{\xi ,n}^{2}}\right){\left(\int_{\Delta }{\hat{\beta }}_{n}\left(\delta \right)dQ\left(\delta \right)\right)}^{2}\overset{A}{∼}X_{1}^{2}$$

under $H_{0}^{\prime \prime }$. By contrast, under $H_{1}^{\prime \prime }$, $a_{*}:=\int_{\Delta }\beta_{*}\left(\delta \right)dQ\left(\delta \right)\neq 0$, so that (A1) implies that

$$\left(\frac{{\hat{\sigma }}_{u,n}^{2}}{{\hat{\sigma }}_{\xi ,n}^{2}}\right){\left(\sqrt{n}\int_{\Delta }{\hat{\beta }}_{n}\left(\delta \right)dQ\left(\delta \right)-\sqrt{n}a_{*}\right)}^{2}\overset{A}{∼}X_{1}^{2},$$

where the left side is asymptotically identical to

$$W_{n}-2n\left(\frac{{\hat{\sigma }}_{u,n}^{2}}{{\hat{\sigma }}_{\xi ,n}^{2}}\right)a_{*}\left(\int_{\Delta }{\hat{\beta }}_{n}\left(\delta \right)dQ\left(\delta \right)-a_{*}\right)-n\left(\frac{{\hat{\sigma }}_{u,n}^{2}}{{\hat{\sigma }}_{\xi ,n}^{2}}\right)a_{*}^{2},$$

so that $W_{n}=n\left({\hat{\sigma }}_{u,n}^{2}/{\hat{\sigma }}_{\xi ,n}^{2}\right)a_{*}^{2}+O_{P}\left(\sqrt{n}\right)$, and this implies that $W_{n}/n=\left(\sigma_{u}^{2}/\sigma_{\xi }^{2}\right)a_{*}^{2}+o_{P}\left(1\right)$ under $H_{1}^{\prime \prime }$ from the fact that both ${\hat{\sigma }}_{u,n}^{2}$ and ${\hat{\sigma }}_{\xi ,n}^{2}$ are consistent for ${\hat{\sigma }}_{u}^{2}$ and ${\hat{\sigma }}_{\xi }^{2}$, respectively. Therefore, for any $c_{n}=o\left(n\right)$, $\lim_{n\to \infty }P\left(W_{n}>c_{n}\right)=1$ as desired. (*ii*) Note that

$$W_{m,n}:=n{\hat{\sigma }}_{n}^{2}\left(\frac{{\hat{\xi }}_{m,n}^{2}}{{\hat{\sigma }}_{\xi ,m,n}^{2}}\right),$$

where

$$\left[\begin{array}{c} {\hat{\gamma }}_{m,n}\\ {\hat{\xi }}_{m,n} \end{array}\right]:={\left[\begin{array}{cc} 1 & \sum_{t=1}^{n}{\hat{u}}_{t}\\ \sum_{t=1}^{n}{\hat{u}}_{t} & \sum_{t=1}^{n}{\hat{u}}_{t}^{2} \end{array}\right]}^{-1}\left[\begin{array}{c} \sum_{t=1}^{n}{\bar{\Psi }}_{t,m}\\ \sum_{t=1}^{n}{\hat{u}}_{t}{\bar{\Psi }}_{t,m} \end{array}\right],\;\;\;\;{\hat{\sigma }}_{\xi ,m,n}^{2}:=\frac{1}{n}\sum_{t=1}^{n}{\hat{u}}_{t}^{2}{\bar{\Psi }}_{t,m,n}^{*2},$$

$${\bar{\Psi }}_{t,m,n}^{*}:={\bar{\Psi }}_{t,m}-\left(\frac{1}{n}\sum_{t=1}^{n}{\bar{\Psi }}_{t,m}w_{t}\left(p\right)\right){\left(\frac{1}{n}\sum_{t=1}^{n}w_{t}\left(p\right)w_{t}{\left(p\right)}^{\prime }\right)}^{-1}w_{t}\left(p\right),\;\;\;\mathrm{and}$$

$${\bar{\Psi }}_{t,m}:=\frac{1}{m}\sum_{i=1}^{m}\Psi_{t}\left(\delta_{i}\right).$$

Therefore, if for each *t*, ${\bar{\Psi }}_{t,m}\overset{a.s.}{\to }\int_{\Delta }\Psi_{t}\left(\delta \right)Q\left(\delta \right)$, it follows that as m→∞, ${\hat{\xi }}_{m,n}\overset{a.s.}{\to }{\hat{\xi }}_{n}$ and ${\hat{\sigma }}_{\xi ,m,n}^{2}\overset{a.s.}{\to }{\hat{\sigma }}_{\xi ,n}^{2}$, so that $W_{m,n}\to W_{n}$ as m→∞, and the desired result follows from Lemma 1(*i*). The law of large numbers can apply to ${\bar{\Psi }}_{t,m}$, so that as m→∞, ${\bar{\Psi }}_{t,m}\overset{a.s.}{\to }E_{Q}\left[\Psi_{t}\left(\delta_{i}\right)\right]$ from the fact that $\delta_{i}$ is drawn from Q(·) and independent of the data observations. Furthermore, $E_{Q}\left[\Psi_{t}\left(\delta_{i}\right)\right]=\int_{\Delta }\Psi_{t}\left(\delta \right)Q\left(\delta \right)$ by the definition of the expectation. This completes the proof.  ☐

**Proof** **of Corollary 1.** For notational simplicity, we let $W_{m,n}\left(p\right)$ denote the WELM test statistic testing $M_{p}^{0}$ against $M_{p}$. From the definition of ${\hat{p}}_{n}\left(\alpha \right)$, namely

$${\hat{p}}_{n}\left(\alpha \right):=\arg\min\left\{P:W_{m,n}\left(p\right)\leq cv\left(\alpha \right)\right\},$$

where cv(α) is the critical value obtained from the chi-squared distribution with one degree of freedom and level of significance α, if $p<p_{*}$,

(A2) $$\lim_{n\to \infty }P\left({\hat{p}}_{n}\left(\alpha \right)=p\right)=0,$$

because if $p<p_{*}\in P$, the model is misspecified, so that for any positive sequence $\left\{c_{n}\right\}$ such that $c_{n}=o\left(n\right)$,

(A3) $$P(W_{m,n}\left(p\right)>c_{n})\to 1$$

as n→∞. Therefore, it follows that

$$\lim_{n\to \infty }P\left(I\left(p=p_{*}\right)|W_{m,n}\left(p\right)\right)=0,$$

where $P(I\left(p=p_{*}\right)|W_{m,n}\left(p\right))$ denotes the conditional probability for the hypothesized polynomial degree *p* being equal to $p_{*}$ conditional on that the hypothesis $M_{p}^{0}$ is tested by $W_{m,n}\left(p\right)$, implying (A2). Therefore,

(A4) $$\lim_{n\to \infty }P\left({\hat{p}}_{n}\left(\alpha \right)\geq p_{*}\right)=1.$$

By contrast, if $p\geq p_{*}$, the model is correctly specified and $W_{m,n}\left(p\right)\overset{A}{∼}X_{1}^{2}$ from the structure of the WELM test statistic, so that

$$P(W_{m,n}\left(p\right)>cv\left(\alpha \right))\to \alpha$$

as n→∞. That is, it follows that for each $p\geq p_{*}$,

(A5) $$\lim_{n\to \infty }P\left(I\left(p=p_{*}\right)|W_{m,n}\left(p\right)\right)=1-\alpha .$$

Therefore, the definition of ${\hat{p}}_{n}\left(\alpha \right)$, (A4), and (A5) imply that ${\hat{p}}_{n}\left(\alpha \right)$ consistently estimates the minimum value of $\left\{p\in P:p\geq p_{*}\right\}$ with probability 1−α, which is $p_{*}$. This implies the desired result.  ☐

**Proof** **of Theorem 1.** Let $cv_{n}$ be the critical value corresponding to $\alpha_{n}$, namely

$$cv_{n}=C^{-1}\left(1-\alpha_{n}\right),$$

which is $O(n^{\delta })$ and also o(n) because δ∈(0,1) by the given condition. Therefore, for each *p*, (A3) implies that

$$P(W_{m,n}\left(p\right)>cv_{n})\to 1,$$

implying that

(A6) $$\lim_{n\to \infty }P\left({\hat{p}}_{n}\left(\alpha_{n}\right)\geq p_{*}\right)=1.$$

Contrary to this, if $p\geq p_{*}$, $W_{m,b}\left(p\right)\overset{A}{∼}X_{1}^{2}$, so that

$$\lim_{n\to \infty }P\left(W_{m,n}\left(p\right)>cv_{n}\right)-\alpha_{n}=0,$$

and $\alpha_{n}=o\left(1\right)$ because $cv_{n}=O\left(n^{\delta }\right)$ for some δ>0. Therefore, for each $p\geq p_{*}$, it follows that

(A7) $$\lim_{n\to \infty }P\left(W_{m,n}\left(p\right)>cv_{n}\right)=0,$$

and this and (A6) imply that

$$\lim_{n\to \infty }P\left({\hat{p}}_{n}\left(\alpha_{n}\right)=p_{*}\right)-\left(1-\alpha_{n}\right)=0,$$

because ${\hat{p}}_{n}\left(\alpha_{n}\right)$ is defined to be the smallest degree among the degrees satisfying (A7). The final equation now implies that $P\left({\hat{p}}_{n}\left(\alpha_{n}\right)=p_{*}\right)=1+o\left(1\right)$. This completes the proof.  ☐
